# Rapid Identification of Genetic Modifications in *Bacillus anthracis* Using Whole Genome Draft Sequences Generated by 454 Pyrosequencing

**DOI:** 10.1371/journal.pone.0012397

**Published:** 2010-08-25

**Authors:** Peter E. Chen, Kristin M. Willner, Amy Butani, Shakia Dorsey, Matroner George, Andrew Stewart, Shannon M. Lentz, Christopher E. Cook, Arya Akmal, Lance B. Price, Paul S. Keim, Alfred Mateczun, Trupti N. Brahmbhatt, Kimberly A. Bishop-Lilly, Michael E. Zwick, Timothy D. Read, Shanmuga Sozhamannan

**Affiliations:** 1 Naval Medical Research Center, Biological Defense Research Directorate, Silver Spring, Maryland, United States of America; 2 Translational Genomics Research Institute, Flagstaff, Arizona, United States of America; 3 Northern Arizona University, Flagstaff, Arizona, Unites States of America; 4 Department of Human Genetics, Emory University School of Medicine, Atlanta, Georgia, United States of America; 5 Division of Infectious Diseases, Department of Medicine, Emory University School of Medicine, Atlanta, Georgia, United States of America; Deutsches Krebsforschungszentrum, Germany

## Abstract

**Background:**

The anthrax letter attacks of 2001 highlighted the need for rapid identification of biothreat agents not only for epidemiological surveillance of the intentional outbreak but also for implementing appropriate countermeasures, such as antibiotic treatment, in a timely manner to prevent further casualties. It is clear from the 2001 cases that survival may be markedly improved by administration of antimicrobial therapy during the early symptomatic phase of the illness; i.e., within 3 days of appearance of symptoms. Microbiological detection methods are feasible only for organisms that can be cultured *in vitro* and cannot detect all genetic modifications with the exception of antibiotic resistance. Currently available immuno or nucleic acid-based rapid detection assays utilize known, organism-specific proteins or genomic DNA signatures respectively. Hence, these assays lack the ability to detect novel natural variations or intentional genetic modifications that circumvent the targets of the detection assays or in the case of a biological attack using an antibiotic resistant or virulence enhanced *Bacillus anthracis*, to advise on therapeutic treatments.

**Methodology/Principal Findings:**

We show here that the Roche 454-based pyrosequencing can generate whole genome draft sequences of deep and broad enough coverage of a bacterial genome in less than 24 hours. Furthermore, using the unfinished draft sequences, we demonstrate that unbiased identification of known as well as heretofore-unreported genetic modifications that include indels and single nucleotide polymorphisms conferring antibiotic and phage resistances is feasible within the next 12 hours.

**Conclusions/Significance:**

Second generation sequencing technologies have paved the way for sequence-based rapid identification of both known and previously undocumented genetic modifications in cultured, conventional and newly emerging biothreat agents. Our findings have significant implications in the context of whole genome sequencing-based routine clinical diagnostics as well as epidemiological surveillance of natural disease outbreaks caused by bacterial and viral agents.

## Introduction

The central challenge in the rapid detection, identification and characterization of microbial pathogens lies in the accurate recognition of a trait, or combination of traits, that is unique to a specific bacterial strain [1], [2]. Traditional laboratory methods largely used different types of phenotypic assays to perform this important task, although this approach is limited to organisms that can be cultured in a laboratory. Increasingly, DNA based assays that detect known genomic signatures have been developed that offer rapid and reliable identification of microbial pathogens [3], [4]. These approaches can be used with both non-culturable pathogens and when sample quantity is limiting. However, both the traditional phenotypic and more recent DNA-based assays suffer from a common limitation. They both require prior knowledge of specific genetic variants that are found only within the known microbial pathogen and are never found in unrelated organisms. As a consequence, newly arising microbial strains or species with functionally important, but previously unobserved, genomic variants may prove difficult or impossible to detect and identify.

The ongoing revolution in DNA sequencing [5], [6] enabling ever-increasing sequence production at an ever-decreasing cost per base, offers an opportunity to relax the requirement for prior knowledge of strain-specific variants. Furthermore, the relatively small footprint, both in terms of laboratory space and personnel, required by these technologies may in the future enable them to be broadly available for a large number of detection and identification applications. New DNA sequencing platforms are already enabling novel approaches to characterize bacterial genomes [7], [8], [9], [10], while at the same time profoundly altering our understanding of the natural genetic variation present in microbial populations [11], [12].

*Bacillus anthracis*, a category A biothreat agent, is a spore-forming, Gram-positive bacterium of the *Bacillus cereus sensu lato* group. It is the etiologic agent of anthrax, which is primarily a zoonotic disease associated with livestock. Until the intentional anthrax attacks in October and November 2001, human cases of inhalational anthrax in the United States have been historically associated with workers in textile and tanning industries while two cases of anthrax-like disease have been described in welders infected by *B. cereus* [13], [14], [15]. Over a period of 100 years (1900–2000) in the U.S. there were 18 recognized cases of inhalational anthrax with a fatality rate (16/18) of 85% [15], [16]. In Sverdlovsk, former Soviet Union, in 1979, there was an outbreak of anthrax cases due to accidental release of anthrax spores from a biowarfare production facility, with similarly high fatality rate of 86% (68/79) although the number of cases was unclear and the mortality rate is probably lower [17], [18]. The recent U. S. anthrax attack resulted in 11 confirmed cases with five deaths -fatality rate 45% of inhalational anthrax and seven confirmed and four suspected cases of cutaneous anthrax all of whom survived [18].

**Figure 1:**
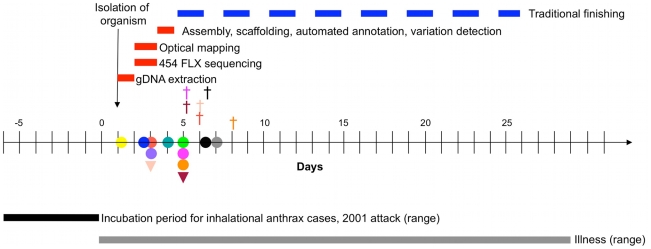
Estimated timelines of WGS and finishing in relation to anthrax disease progression in anthrax cases. The anthrax disease progression and the results of antibiotic treatment in the first ten cases of the 2001 anthrax letter attack [13] and two cases of anthrax-like disease caused by *B. cereus* in 1996 [14]. Colored circles indicate when individual cases of inhalational anthrax from 2001 attack were started on antibiotic therapy and colored triangles indicate when 1996 *B. cereus* fatal pneumonia cases resembling anthrax were started on antibiotic therapy. The crosses indicate the time of death of 6 of the 12 patients. The bars in the top part of the top panel indicate the estimated timelines involved in obtaining WGS sequencing from the time the patient seeks medical care. Currently, there is no standard protocol or fixed time frame for genome finishing and the process varies from a few months to years depending on the complexity of the genome and approaches used.

Some of the epidemiological and clinical features of the 2001 U.S. cases have been published [13]. In 20 of the 22 cases a clear link to letters containing anthrax spores was established [18]. Figure 1 summarizes the timeline of disease progression in the first ten of the 2001 inhalational anthrax cases and two cases of anthrax-like disease caused by *B. cereus* in 1996 [13], [14], in relation to the onset of symptoms and antibiotic therapy. In the first ten cases of 2001 inhalational anthrax, the median incubation period from the time of exposure to onset of symptoms when known (n = 6) was 4 days (range 4 to 6 days) and the mean was 4.5 days. Victims sought care a median of 3.5 days (range 1 to 7 days) after onset of symptoms. Eight of ten patients were in the early symptomatic phase of illness when they first sought care. Of these eight, six received antibiotics active against *B. anthracis* on the same day, and all six survived. The two 1996 patients and four of the 2001 patients were exhibiting fulminant signs of illness when they first received antibiotics and all six died. It is clear from these cases that the survival may be markedly improved by administration of antimicrobial therapy during the early symptomatic phase of the illness; i.e., within the first 3 days of appearance of symptoms. All patients received combination antimicrobial therapy with more than one agent active against *B. anthracis*, which may have contributed to the apparent improvement in survival compared with previous cases. Other possible explanations for the improved survival rate may include better supportive care, differences in the pathogenesis of bioterrorism-related anthrax, differences in susceptibility of the hosts, or a combination of the above [13]. Thus, early recognition of the pathogen and deciphering its virulence properties, antibiotic susceptibility profile and any evidence of genetic modifications natural or intentional are critical challenges facing the biodefense community. Conventional methods lack the ability to decipher all these properties in a clinically relevant time frame.

In the last nine years since the 2001 anthrax cases, considerable effort has been expended in developing rapid diagnostics and therapeutics for biodefense agents [19]. Recent advances in second generation sequencing technologies have allowed us to take this one step further: sequence-based identification of pathogens and of their virulence characteristics. Several recent studies have demonstrated the potential of second generation sequencing technologies for linking a phenotype to a specific genotype [8], [20], [21], [22]. A recent study reported the elegant use of second generation sequencing technology for epidemiological tracking and documenting the evolution of methicillin resistant *Staphylococcus aureus* (MRSA) during hospital transmission and intercontinental spread [23]. However, none of these studies have addressed the timeline needed for deciphering genetic variants in a rapid response scenario. An earlier attempt to predict the timeline involved in deciphering genetic changes in a clinical context proposed a six-week time frame for *Francisella tularensis* using whole-genome sequencing (WGS) [24]. We reasoned that this time frame could be shortened considerably in a rapid response scenario, at least in the case of *B. anthracis*, using current technologies and bioinformatic tools.

In this work we used currently available laboratory technologies and bioinformatic tools and applied them in a number of different scenarios, with a particular emphasis on how to rapidly recognize genetic modifications in a known biodefense pathogen, thus defining the time frame for this process. Using avirulent erythromycin [25] and ciprofloxacin resistant *B. anthracis* strains [26], we tested whether the genetic changes conferring antibiotic resistance can be deciphered rapidly and accurately using WGS. We demonstrate the utility of Roche 454 pyrosequencing technology [27] and a bioinformatic pipeline to rapidly map both known and previously unreported insertion, deletion and point mutations conferring antibiotic and phage resistances in this organism. Our work provides a proof of principle demonstrating the potential of whole genome sequencing for the rapid identification of relevant genetic variants in the public health and biodefense arenas.

## Results

### 454 pyrosequencing of *B. anthracis* wild type and mutant derivatives

We resequenced and annotated eight *B. anthracis* genomes in order to test the accuracy of 454 WGS technology for rapid detection of genetic modifications in this organism. The bacterial strains sequenced in this study and some of the basic statistics on the draft sequence runs are presented in Table 1. All the strains except *gerH::ery* were sequenced once either by FLX or Titanium, and as expected the average read lengths were longer in Titanium runs (361±26 bps) than FLX runs (264±15 bps). The number of contigs for each *de novo* assembly was proportional to the percentage of Q39 or lower quality bases (Table 1). The ΔANR and HS-2-1 genomes assembled into a relatively higher number of contigs compared to the other assemblies due to the contribution of their higher proportion of lower (≤Q39) quality bases. Lower quality bases are likely to be sequencing errors that result in contig breakage. Although genome coverage varied from 8x to 45x, the percentage of the reference sequence covered changed by only 0.92%.

**Table 1:** Statistics of genome sequence of wild type and mutant *B. anthracis* strains.

Strain	Reference Genome	No. runs	Run type	Total no. reads assembled (% all reads)	Average read length (bps)	Length of assembled genome (bps)	No. contigs	Coverage	% ≤Q39	% Refseq covered
Δ**ANR**	Ames Ancestor	1	Titanium	138,842 (97.08)	355	5,071,481	1015	10	2.68	98.52
**HS-2-1**	Ames Ancestor	1	Titanium	109,279 (96.50)	355	5,046,834	1597	8	3.34	98.25
**HS-2-5**	Ames Ancestor	1	FLX	238,564 (99.46)	246	5,169,109	141	11	0.29	99.04
**HS-2-5-6**	Ames Ancestor	1	FLX	378,274 (99.63)	252	5,172,491	85	18	0.06	99.03
**34F2**	Sterne	1	FLX	453,716 (99.54)	274	5,356,131	101	23	0.06	99.07
**gerH::ery**	Sterne	2	FLX	708,078 (99.84)	265	5,357,184	85	35	0.06	99.06
**gerH::ery**	Sterne	1	Titanium	598,555 (98.68)	398	5,357,787	106	45	0.13	99.17
**AP50R**	Sterne	1	FLX	398,980 (99.75)	282	5,355,287	104	21	0.06	99.05

#### Testing the timeline involved in 454 based WGS and identification of genetic modifications Correct identification of previously known erythromycin cassette insertion in *B. anthracis*

We directly tested whether the 454 WGS pipeline we propose can generate reliable genome sequence data in the narrow window of opportunity (3.5 days) presented by the timelines of anthrax disease progression in the victims of 2001 anthrax letter attacks (Figure 1). To test the timeline, we used *B. anthracis* Sterne (34F2) strain and its derivative, *gerH::ery* mutant [25] for WGS either by FLX or Titanium protocols.

**Figure 2:**
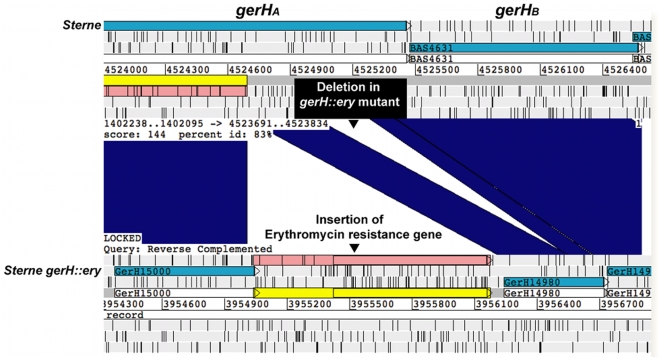
Alignment of *gerH* region between 34F2 and *gerH::ery* mutant. Screenshot of the *gerH* region of *B. anthracis* strains 34F2 and *gerH::ery* mutant by Artemis comparison tool (ACT)-based genome comparison. Blue bars indicate regions sharing significant matches using the BLAST alignment tool. The genome viewer tool shows the insertion of the erythromycin gene in the *gerH_A_* gene and a small deletion within *gerH_A_* gene downstream of the insertion.

Figure 1 also illustrates the timeline for WGS using Roche 454 pyrosequencing technology for comparison. Starting with purified genomic DNAs of strains 34F2 and *gerH::ery* mutant we obtained the draft sequences of each of these strains in 24 hours using the FLX protocol. By comparison, sequencing using the Titanium protocol took about 30 hours, because of longer bead washing and signal processing steps. *De novo* sequence assembly took approximately 45 minutes per genome and, notably, annotation was completed within 40 minutes to 4 hours using a Linux 10-node cluster, whereas performing this same annotation on a desktop computer could take 24 to 48 hours. Three parallel approaches were undertaken to identify the erythromycin resistance cassette in the draft sequences of the *gerH::ery* mutant. 1) The contigs from the mutant's genome were aligned and compared to the annotated reference genome (*B. anthracis* Sterne) by BLASTN using Artemis Comparison Tool (ACT) [28] to identify the genetic modification in the *gerH* gene. This comparison correctly identified an approximately 1.2 kb insertion at the *ClaI* site in the *gerH_A_* gene of the mutant. This 1.2 kb sequence was correctly identified as the erythromycin cassette used in the original study [25]. This analysis also revealed a 265 bp deletion downstream of the erythromycin insertion (Figure 2). 2) Comparison of the genome sequences of 34F2 and the *gerH::ery* mutant by UniqueMER analysis and subsequent BLAST analysis identified the same inserted bases. 3) BLAST analysis of the unique genes from DIYA output of the mutant's sequence against ARDB (antibiotic resistance genes database) [29] identified an erythromycin resistance gene not present in its parent strain 34F2. In addition to this insertion, we also found a high confidence variation in the *gerH* mutant at *BAS2094* that encodes a heat shock protein of the *hsp20* family. The relevance of this mutation in spore germination negative phenotype is not clear at this time. Thus, starting with pure genomic DNA, within 36 hours we identified the insertion of the erythromycin cassette in the *gerH* gene.

#### Correct identification of previously known point mutations in quinolone resistance-determining regions (QRDRs) conferring ciprofloxacin resistance in *B. anthracis*

Next we asked whether we could identify point mutations conferring antibiotic resistance in *B. anthracis* by WGS. For two of the four medium-level ciprofloxacin resistant strains sequenced in this study (HS-2-1 and HS-2-5), mutations had been mapped in a prior study [26]. HS-2-1 is resistant to medium-level ciprofloxacin, [minimal inhibitory concentration (MIC): 8 ug/ml] and carries mutations in *gyrA* (C->T) and *parC* (G->A) genes, while strain HS-2-5 carries the same *gyrA* mutation (C->T) and a different *parC* mutation (G->T). We tested whether one can identify these known mutations rapidly and accurately in low-coverage (8x and 11x for HS-2-1 and HS-2-5, respectively) whole genome draft sequences. Using the GS Reference Mapper tool to map reads against the reference genome sequence of Ames Ancestor (NC 007530), we correctly identified the known mutations in the two ciprofloxacin resistant strains (Table 2).

**Table 2:** Location of true positive variants in the genomes of mutant *B. anthracis* compared to the reference genome.

Strain	Parent strain	Start position	Stop position	Reference allele	Sample allele	Locus description	Coverage	% Concordance
**HS-2-1**	ΔANR	6,848	6,848	C	T	gyrA	5	100%
		3,364,887	3,364,887	G	A	parC	5	100%
		4,009,233	4,009,233	G	A	Stage 0 sporulation protein A	6	100%
		4,373,941	4,373,941	C	A	Hypothetical protein GBAA4810	6	100%
**HS-2-5**	ΔANR	6,848	6,848	C	T	gyrA	10	100%
		2,667,930	2,667,930	T	-	Hypothetical protein GBAA2880	14	100%
		3,364,887	3,364,887	G	T	parC	9	100%
		4,500,491	4,500,491	G	A	intergenic	9	100%
**HS-2-5-6**	HS-2-5	841,481	843,099	1618 bp	Δ	tetR family transcriptional regulator	0 (Δ)*	100%
		3,588,786	3,588,786	T	C	phosphodiesterase	14	100%
		4,009,850	4,009,873	24 bp	Δ	Stage 0 sporulation protein A	11	100%
		4,047,733	4,047,733	G	T	Hypothetical protein GBAA4446	21	100%
**gerH::ery** **	34F2 chr	4,524,685	4,524,685	-	EryR	gerH_A_	45	100%
		2,099,226	2,099,226	T	C	HSP20 family protein	33	100%
	pXO1	172,178	172,178	20 bp	Δ	none	66	95%
**AP50R**	34F2 chr	895,066	895,066	A	-	csaB	14	100%
		3,894,626	3,894,626	G	A	2,3-diketo-5-methylthiopentyl-1-phosphate enolase	8	100%

*coverage for the 5′ base (841,480) flanking the deletion was 13x and the 3′ base (843,100) flanking the deletion was 4x.

**includes both FLX and Titanium runs.

Identification of previously undocumented putative variations

Having shown the utility of WGS for mapping known changes (insertion and point mutations), we tested whether we could identify previously unknown genotypes for specific phenotypes. For two other strains sequenced in this study, the genetic changes responsible for the phenotypes (high-level ciprofloxacin resistance and phage AP50c resistance) were not known prior to this work and we wanted to test whether one can identify the putative loci responsible for these phenotypes from the draft sequences.

#### Identification of genetic changes leading to high-level ciprofloxacin resistance

Based on prior published data, strain HS-2-5-6 exhibited high-level ciprofloxacin resistance (MIC: 32 ug/ml). Sanger sequencing of the *gyrA* and *parC* genes had revealed identical mutations to HS-2-1 but no additional mutations were found in any of the known QRDR *loci*. In addition, the involvement of an efflux pump had been implicated by increased susceptibility to ciprofloxacin in the presence of the potent efflux pump inhibitor INF271 [26]. However, the gene responsible for the efflux pump was not known prior to this work. From single nucleotide polymorphism (SNP) and indel analyses, four high-confidence variations were found to be unique to this strain (Table 3). Of these, a deletion of 1,618 base pairs in an ORF encoding a TetR-family transcriptional regulator (*GBAA0834*) appears to be the most probable candidate for high-level ciprofloxacin resistance. Four lines of evidence support this idea: a) the ORF lies upstream of a gene that encodes a drug efflux pump; b) other high-level ciprofloxacin mutants have been found in this study to carry similar mutations in this ORF (data not shown); c) these mutations were confirmed to be true positive variations by PCR analyses (data not shown); d) during the preparation of this manuscript another study reported the involvement of this gene in high-level ciprofloxacin resistance [21]. The precise mechanism by which mutations in this gene lead to high-level ciprofloxacin resistance is currently under investigation in our lab. Thus, using draft, unfinished sequences produced by WGS we identified a novel deletion potentially conferring high-level ciprofloxacin resistance.

**Table 3:** Determination of putative variants in the genomes sequenced in this study.

Strain	No. putative variants (LQ + HQ)	No. HQ putative variants	No. unique HQ putative variants*	No. Sanger verified unique HQ putative variants (TP)
ΔANR	71	1	1	1
HS-2-1	150	18	17	4
HS-2-5	23	5	4	4
HS-2-5-6	44	8	4	3
34F2	101	48	48	39
gerH::ery **	425	97	17	1
AP50R	65	44	2	2

LQ Low quality. HQ High quality. TP True positive.

*Does not include identity by descent variants from descendant strains.

**Includes both FLX and Titanium runs.

#### Identification of a genetic change leading to spontaneous phage resistance

Strain 34F2_AP50R was isolated as a survivor of AP50c phage infection of *B. anthracis* strain 34F2. Wild type 34F2 is susceptible to infection by AP50c phage but survivors of phage infection appeared in a population at very low frequencies [30]. This mutant was used for mapping the mutation causing phage resistance. Whole genome sequencing of 34F2_AP50R was performed using Roche/454 FLX protocol.

When the 34F2_AP50R WGS data were compared to the parent 34F2, we found two high confidence variations in the phage resistant mutant (Table 3). We reasoned that, of the two, *csaB* (*BAS0840*) was the most probable candidate for phage resistance since it is located in an operon just upstream of the *sap* gene, which encodes an S-layer protein that is on the surface of the bacterium [31]. Furthermore, we constructed a targeted deletion and a number of point mutants of the *csaB* gene by markerless gene replacement method described earlier [32] and these mutants were found to result in AP50c phage resistance (manuscript in preparation). We have shown that indeed the *sap* gene is also involved in phage resistance and most likely is the receptor of AP50c phage by creating a targeted deletion of this gene and showing that the *Δ sap* strain is phage resistant (manuscript in preparation).

### All other variations found in the draft sequences

**Figure 3:**
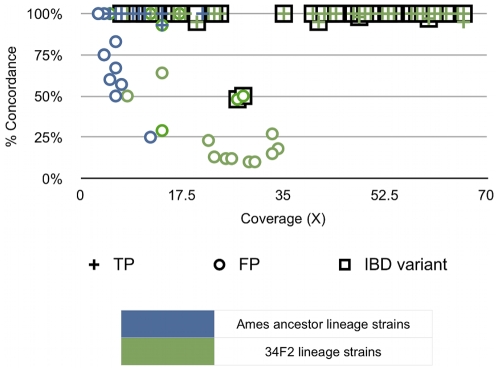
Sanger-verified true positive variations as a function of percent concordance and coverage. Validation of high quality variations in the genome sequences produced by GS Reference Mapper software. The graph shows the percentage of concordance among the reads covering a variant position as a function of depth of coverage. The graph indicates all the high-quality unique variations in the eight genomic sequences obtained in this work. Circles represent false positive variations while plus signs indicate true positive variations as determined by Sanger sequencing. Squares represent identity-by-descent (IBD) variations that were not verified by Sanger sequencing as they were expected to be present in the descendant strains. The various colors of the symbols represent different strains.

All other variations were evaluated by the following scheme in order to determine the possible true positive versus false positive variations. The GS Reference Mapper algorithm identified 879 SNPs in all the eight sequences obtained in this study. Of these, 221 were high quality (HQ) and 658 were low quality variations (LQ) as defined by the same algorithm (see materials and methods). Of the 221 HQ variations, 32 were found in ΔANR derivatives and 189 in 34F2 and its derivatives. Of the 221 variations, only 93 were unique to any particular strain, while the rest were carried from the parent to the descendants (identity-by-descent variations). Thus, there were 93 unique HQ variations in the seven strains (Table 3). True positive variations among the 93 were determined by two different approaches. Firstly, Figure 3 shows a graph of the percentage concordance (percentage of reads with identical variant sequence) of the variant reads as a function of depth of coverage for the respective position on the genome. We postulated that true positive variations could be largely determined as a function of the concordance level; i.e., variations with very high concordant values would be true positives. Secondly, we conducted a second round of sequencing of the high-quality unique variants using the Sanger method to empirically test this prediction. Sanger verification revealed that all the expected causal variations related to the mutant phenotypes of interest were true positives. In addition Sanger sequencing confirmed as true positive several other variations. The locations of the true positive variations in the *B. anthracis* mutants are listed in Table 2, while the true positive variations in parent strains are listed in Table S1. Overall, only four out of 62 variations with a concordance of ≥85% turned out to be false positive whereas no true positive was found among the 27 variations with <85% concordance. Based on these data, we conclude that the true positive variations have very high concordance rates (≥85%) and most, if not all, low-concordance variations are false positive variations.

## Discussion

Almost nine years after the worst bioterrorism attacks in U.S. history and subsequent extensive efforts in developing rapid diagnostics and therapeutics to tackle such events some questions still remain: How well are we prepared to face a similar attack? How do we measure progress? One of the ways to address these issues is presented in this article; i.e., can we identify a naturally occurring or genetically engineered biothreat agent in a timely manner. Because there was only a single strain responsible for the 2001 anthrax outbreak, decisions regarding initial treatment could be made empirically [18]. However, isolation of genetically engineered or spontaneous antibiotic resistant *B. anthracis* strains has been reported in the past [33], [34]. The implication is that selection of antibiotic regimen was dictated for this event and will be for any future episodes by the results of *in vitro* susceptibility testing of the implicated strain [18]. Unlike rapid antibiotic susceptibility testing, empirical determination with regard to altered or novel virulence cannot be obtained in 72 hours. We reasoned that, if WGS were used, this narrow window of opportunity (72 hours) might be enough for deciphering genetic modifications altering antibiotic susceptibility as well as virulence in the suspected organism. We demonstrate that at least for a monomorphic pathogen that can be cultured quickly, it is indeed possible to identify both known and previously unknown genetic modifications as rapidly as 60 hours using one of the second generation sequencing technologies (454 pyrosequencing).

Another compelling reason for whole-genome sequencing-based identification of genetic modifications is the emergence of novel pathogens causing anthrax-like disease. Simple diagnostic tests based on *B. anthracis* markers have the potential to exclude these organisms as non-*anthracis* isolates, while sequencing can reveal the presence of virulence factors similar to those present in *B. anthracis* [35], [36], [37].

We demonstrate in this work that we could identify known insertion and point mutations conferring antibiotic resistance in *B. anthracis*. We also deciphered a heretofore-unreported putative gene (*GBAA0834*) conferring high-level ciprofloxacin resistance. Taking a similar approach, Serizawa *et al* identified the same gene using short read sequence data generated by the Illumina Genome Analyzer II sequencer [21]. The alleles of *GBAA0834* described by these authors are mostly SNVs (single nucleotide variations) whereas we have found large deletions in five of 17 additional high-level ciprofloxacin resistant mutants screened by PCR of *GBAA0834* (HS-2-1-1, HS-2-1-4, HS-2-1-7, HS-2-1-8, and S3-5; data not shown). Further characterization of the remaining mutants is underway in our lab. The mechanism by which inactivation of the *tetR* type regulatory gene leads to high-level ciprofloxacin resistance is not entirely clear at present; it may lead to derepression of the expression of the downstream gene, which encodes a drug efflux pump. In support of this hypothesis, Serizawa *et al.* [21] found in their mutant strains upregulation of all three genes (*GBAA0832*, *GBAA0833* and *GBAA0835*) adjacent to the TetR type regulator (*GBAA0834*). However, based on the deletion endpoints found in strain HS-2-5-6, (which spans *GBAA0832* and *GBAA0833*) we speculate that it is the expression of *GBAA0835* that is responsible for high-level ciprofloxacin resistance while the other two genes are not involved in this phenotype.

**Figure 4:**
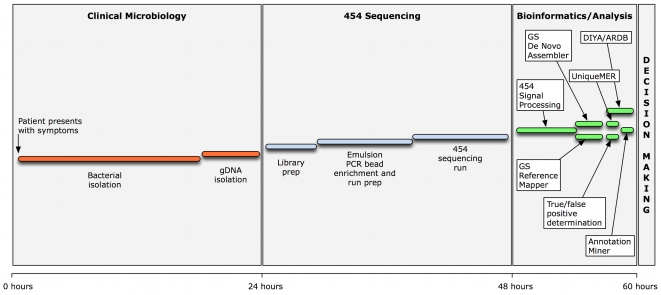
Proposed pipeline for rapid identification of genetic modifications in *B. anthracis*. The three stages involved in genetic characterization of *B. anthracis* in a rapid response scenario. The first day is needed for isolation of the organism from the patient and extraction of genomic DNA. During the second day WGS will be performed using Roche 454 technology. The third day is needed to analyze the genome sequence data and figure out genetic modifications. The various bioinformatics tools used in this work are shown.

Figure 4 shows the general outline of the pipeline we adopted to sequence and identify genetic modifications in *B. anthracis*. We estimated that within 24 hours after the patient seeks medical care, the suspected bacterium can be isolated and its genomic DNA extracted. We demonstrated that in the next 36 hours WGS can be performed and the resulting draft sequences can be analyzed to pin down any genetic modification in the suspected organism using existing bioinformatic tools. The actual timeline involved in these processes may vary and will depend on the complexity and culturability of the organism and computing capacity of the facility. The organism used in this study, *B. anthracis*, is known to be a highly monomorphic species. On the other hand, the genome from an organism with repeat regions or highly variable sequences may show a much higher number of variations compared to its most closely related reference genome and hence prove difficult to assemble and take longer than 12 hours for downstream bioinformatic analyses. The process and timeline outlined here may also vary with the type of genetic modifications. For example, we have not looked at inversions and copy number variations or variations in gene expression leading to phenotypic changes or complex polygenic traits. Despite these caveats, it is still feasible to finish WGS of the suspected organism for further analysis within the time frame of 36 hours after isolating the suspected organism. Even in the case of a new organism without a preexisting reference sequence, this time frame would be enough to at least identify the pathogen at the genus and species level and possibly identify any virulence and antibiotic markers by alignment to reference databases. Expanding the repertoire of high quality reference genome sequences of strains belonging to a multitude of genera and species will undoubtedly fill this void.

The time required to perform rapid genome sequencing will continue to shrink as new sequencing platforms, wet lab techniques and bioinformatic analysis pipelines evolve. Metagenome sequencing, which involves sequencing directly from a sample without culturing or purifying the organism, could shorten the timeframe for rapid sequencing in response to a bioterrorism event. There have been several recent examples where this technique has been applied for the purpose of discovering the etiological agent of an outbreak [38], [39], [40], [41]. In one such example, 454 pyrosequencing was performed directly from RNA extract of serum and tissues of hemorrhagic fever victims and detected a novel Lujovirus as the causative agent [41]. However, metagenome sequencing of bacterial pathogens in clinical samples has not yet become an accepted technique for identification of important genetic variants such as drug resistance SNPs.

Our results show that next generation sequencing platforms such as 454 pyrosequencing hold much promise for unbiased and rapid detection, diagnosis, and genomic characterization in the event of a bioterrorism attack or a natural disease outbreak. These platforms deserve further exploration to determine how best to utilize them during such events.

## Materials and Methods

### Bacterial strains

The bacterial strains sequenced in this study are listed in Table 1 and are as follows: *B. anthracis* Sterne strain (34F2, pXO1+, pXO2-); a 34F2 derivative, *gerH::ery*, which has an erythromycin cassette inserted in the *gerH_A_* gene [25] and a 34F2 derivative that confers resistance to phage AP50c due to a unidentified mutation. AP50 is a *B. anthracis* specific diagnostic phage; AP50c is a spontaneous clear plaque mutant [30]. ΔANR is a *B. anthracis* Ames derivative that lacks both pXO1 and pXO2 plasmids [26]. Strain HS-2 is a derivative of ΔANR with low-level ciprofloxacin resistance (MIC): 0.5 ug/ml) due to a mutation in *gyrA* and strains HS-2-1 and HS-2-5 were derived from HS-2 and they exhibit medium-level (MIC: 8 ug/ml) ciprofloxacin resistance due to mutations in the *parC* gene. The high-level ciprofloxacin resistant (MIC: 32 ug/ml) mutant HS-2-5-6 was derived from HS-2-5 strain and it has as yet unidentified genetic changes. The isolation, mapping and characterization of mutants exhibiting medium levels of ciprofloxacin resistance have been published [26].

### Genomic DNA extraction and whole-genome sequencing (WGS) by 454 pyrosequencing

Genomic DNA from various bacterial cultures was extracted using Wizard genomic DNA preparation kit (Promega Inc). Whole-genome sequencing (WGS) was performed using the Genome Sequencer FLX (454 Life Sciences/Roche) using FLX or FLX Titanium reagents according to the manufacturer's protocols and instructions [27]. Signal processing of FLX data was performed on the sequencer itself, whereas signal processing of Titanium data was performed off-rig on a Linux cluster of 10 nodes connected via gigabit ethernet. Each node contained eight 64-bit processing cores running at 2.3 GHz with 8 GB of RAM. *De novo* assembly of either FLX or Titanium sequences, preliminary annotation of the genome using the DIYA pipeline [42] and identification of the erythromycin insertion were performed using the above-described computer cluster. The genome sequences data generated in this study have been deposited in the NCBI database under the project accession number SRP001994.1.

### Assemblies

All genomes were *de novo* assembled using the GS De Novo Assembler (454/Roche). Coverage was defined as the average number of reads covering a site on the genome. ‘% Q39’ is the percentage of bases with a quality score of less than or equal to 39 which is equivalent to an error rate of approximately 1 in 10,000 bases.

### Variation Detection

All putative variants were detected using two software programs, GS Reference Mapper (454/Roche) and UniqueMER (https://sourceforge.net/projects/uniquemer/). A variant flagged by GS Reference Mapper was defined as high quality if it met the following criteria: a) at least three non-duplicated reads showed the same variation; b) at least one read with this variation in both the forward and reverse orientations, unless there are at least five reads with quality scores over 20 (or 30 if the difference involves a 5-mer or higher). A more detailed description of differences is available in the Genome Sequencer Data Analysis Software manual. UniqueMER is a hash-based algorithm that detects unique sequence regions and its approach does not limit the size of indels it can detect.

### Sanger sequence verification of variations found in 454sequences

A total of 95 HQ variations unique to the seven sequenced strains, excluding the identity-by-descent variations were verified by Sanger sequencing. Primers were designed 150–200 bp away from the 5′ and 3′ ends of the variant position and the resulting PCR amplicons (usually 300 to 400 bps) were sequenced using the same primers in an ABI 3700 sequencer. Quality scores were called with the program Phred [43], [44] and the resulting Phred scores along with the reference and parent sequences were visualized using CodonCode Aligner (http://www.codoncode.com/aligner/). Sanger-verified true positive variants showed complete agreement in both forward and reverse orientations and these variants were located within high-quality regions (Phred score ≥20).

### Mapping mutations

The variations were mapped to the respective genes or intergenic regions using the published annotation of the reference genomes Sterne (NC 005945) for 34F2 and its derivatives and Ames Ancestor (NC 007530) for ΔANR and its derivatives by Annotation Miner software (https://sourceforge.net/projects/annotationminer/).

### Genome Annotation

*B. anthracis* contigs resulting from *de novo* assembly were annotated using DIYA (Do-It-Yourself Annotator), a modular and configurable open-source pipeline software written in Perl [42].

## Supporting Information

Table S1 Location of true positive variants in the genome of the parent strains compared to the reference genomes.
(0.09 MB DOC)
