# Supplementary material for: Rapid Identification of Genetic Modifications in Bacillus anthracis Using Whole Genome Draft Sequences Generated by 454 Pyrosequencing
Source: PLoS One. 2010 Aug 25;5(8):e12397. doi: 10.1371/journal.pone.0012397 (PMC2928293; doi:10.1371/journal.pone.0012397)
Supplement: Table S1 — Location of true positive variants in the genome of the parent strains compared to the reference genomes. (0.09 MB DOC) [file pone.0012397.s001.doc]

**Table S1. Location of true positive variants in the genome of the parent strains compared to the reference genomes.**

| **Strain** | **Reference genome** | **Start position** | **Stop position** | **Reference allele** | **Sample allele** | **Locus description** | **Coverage** | **% Concordance** |
| --- | --- | --- | --- | --- | --- | --- | --- | --- |
| Δ **ANR** | Ames ancestor | 4,779,457 | 4,779,457 | G | T | DNA-binding response regulator | 7 | 100% |
| **34F2** | Sterne | 226,566 | 226,566 | G | C | intergenic | 18 | 100% |
|  |  | 629,739 | 629,739 | T | C | iron compound ABC transporter, iron compound-binding protein | 17 | 100% |
|  |  | 876,972 | 876,972 | T | G | HAD superfamily hydrolase | 23 | 100% |
|  |  | 1,373,977 | 1,373,977 | - | G | proton/glutamate symporter protein, N-terminus | 20 | 95% |
|  |  | 1,727,727 | 1,727,727 | G | A | intergenic | 22 | 100% |
|  |  | 1,747,138 | 1,747,138 | G | A | acetyl-CoA hydrolase/transferase family protein | 17 | 100% |
|  |  | 1,962,552 | 1,962,552 | G | - | intergenic | 16 | 100% |
|  |  | 2,076,699 | 2,076,699 | T | C | Alcohol dehydrogenase, iron-containing | 11 | 100% |
|  |  | 3,509, 997 | 3,509,997 | T | C | Hypothetical protein BAS3551 | 13 | 100% |
|  |  | 3,657,937 | 3,657,937 | G | A | tRNA (guanine-N(1)-)-methyltransferase | 12 | 100% |
|  |  | 4,371,853 | 4,371,853 | G | A | Small acid-soluble spore protein SspI | 24 | 100% |
|  | pXO1 | 5,613 | 5,613 | T | - | none | 49 | 100% |
|  |  | 16,527 | 16,527 | G | C | none | 57 | 100% |
|  |  | 31,659 | 31,659 | - | G | none | 35 | 100% |
|  |  | 34,583 | 34,583 | - | T | none | 54 | 100% |
|  |  | 36,499 | 36,499 | G | T | none | 65 | 100% |
|  |  | 36,502 | 36,502 | T | A | none | 66 | 100% |
|  |  | 42,111 | 42,111 | T | C | none | 48 | 100% |
|  |  | 42,147 | 42,147 | T | A | none | 49 | 100% |
|  |  | 63,092 | 63,092 | G | C | none | 50 | 100% |
|  |  | 68,692 | 68,692 | C | G | none | 60 | 100% |
|  |  | 68,980 | 68,980 | C | G | none | 58 | 100% |
|  |  | 74,021 | 74,021 | A | G | none | 44 | 100% |
|  |  | 86,271 | 86,271 | T | - | none | 52 | 100% |
|  |  | 89,588 | 89,588 | - | C | none | 40 | 100% |
|  |  | 90,763 | 90,763 | - | A | none | 63 | 100% |
|  |  | 97,336 | 97,336 | A | - | none | 43 | 100% |
|  |  | 125,172 | 125,172 | G | C | none | 59 | 100% |
|  |  | 125,175 | 125,175 | G | C | none | 61 | 100% |
|  |  | 125,187 | 125,187 | G | C | none | 62 | 100% |
|  |  | 125,201 | 125,201 | G | C | none | 63 | 100% |
|  |  | 138,547 | 138,547 | C | T | none | 60 | 97% |
|  |  | 152,834 | 152,834 | T | G | none | 42 | 100% |
|  |  | 159,324 | 159,324 | - | A | none | 53 | 100% |
|  |  | 160,045 | 160,045 | T | A | none | 48 | 100% |
|  |  | 160,690 | 160,690 | T | - | none | 53 | 100% |
|  |  | 164,504 | 164,504 | A | - | none | 47 | 100% |
|  |  | 172,194 | 172,213 | 20 bp | Δ | none | 41 | 95% |
|  |  | 179,676 | 179,676 | C | T | none | 48 | 100% |
